# Codesigning Solutions for Assistive Technology Service Provision in Queensland, Australia

**DOI:** 10.1111/hex.70322

**Published:** 2025-06-06

**Authors:** Tammy Aplin, Louise Gustafsson, Christy Hogan, Michelle Owens, Hayden Boyd, Tenelle Hodson, Camila Shirota, Michelle Bissett

**Affiliations:** ^1^ The Hopkins Centre Griffith University Brisbane Australia; ^2^ School of Health Sciences and Social Work Griffith University Brisbane Australia; ^3^ Faculty of Health Southern Cross University Gold Coast Australia

**Keywords:** assistive products, codesign, health systems, participatory, patient and public involvement

## Abstract

**Introduction:**

Assistive technology (AT) is an essential element of universal healthcare, with a lack of access to AT a worldwide problem. Collaboration between key stakeholders is essential to understand the provision challenges and to generate possible solutions. In this paper, we describe the involvement of a stakeholder group in a participatory action research process aimed to interrogate and generate solutions for the AT sector in one Australian state.

**Methods:**

Participatory action research with a stakeholder group (*n* = 14) comprising AT users, therapists experienced in providing AT services, representatives from injury or disability support schemes that manage AT provision, AT experts and researchers and representatives from statewide rehabilitation and allied organisations. Four action cycles were conducted to (1) develop the research design and methods, (2) conduct the research and interrogate the findings, (3) generate and prioritise solutions and (4) develop an action plan for the AT sector.

**Results:**

The stakeholder group influenced the design and conduct of a needs assessment (Action Cycles 1 and 2) and collaborated at a solution‐building workshop to generate 10 recommended solutions (Action Cycle 3). In Action Cycle 4, AT users (*n* = 2) in consultation with AT experts (*n* = 2) led the finalisation of solutions and developed the following action plan recommendations: the development of an AT hub, AT mentor training and a training and credentialing system for AT advisors.

**Conclusions:**

Applying participatory action research, with leadership and collaboration from key stakeholders across the AT sector, can enrich processes and outcomes in AT‐related policy research.

**Patient or Public Consultation:**

A stakeholder group was critical to the design and conduct of the needs assessment and was a leader in the collaborative solution generation and prioritisation process, and the development of an action plan.

## 1 Introduction

Access to assistive technology (AT) is a fundamental human right and, for over 2.5 billion people globally, is required in everyday life to participate equitably in society [1, 2]. AT refers to the systems and services related to the delivery of assistive products [3]. This includes a broad spectrum of products for example mobility aids, bathing equipment, communication aids, hearing aids and modifications to the home environment. Access to AT is essential to human well‐being. As outlined in the World Health Organization's Global Report of Assistive Technology [4], to fulfil obligations to the Convention of the Rights of Persons with Disability and to meet the Sustainable Development Goals, nations need to provide effective, affordable and safe AT to all who need it, as part of universal health coverage.

Although access to AT is widely acknowledged as a fundamental human right, across the globe, there are varying levels of AT access from as little as 3% to as high as 90%, with socio‐economic development being a central influencing factor in a country's provision of AT [4]. In Australia, the context of this study, 2.3 million Australians reported using aids or equipment in 2018 and of the 2.5 million Australians with disability, over 40% reported that their needs overall were not fully met [5], and although it is unknown whether this relates to AT, it is likely that at least part of this need may be related to AT need.

The WHO's people‐centred AT ecosystem framework outlines the inherent complexity of the AT sector with coordinated and effective functioning of five interlinked aspects necessary including people, policy, products, provision and personnel [6]. In Australia, one of the key barriers identified is that AT provision is not guided by an overarching framework or policy, leading to inequities [7, 8]. A plethora of funding schemes exist, each with differing policies regarding access to AT and varying eligibility criteria related to age, disability or how the disability was acquired. One of the major funders of AT is the National Disability Insurance Scheme (NDIS), which provides AT to people under 65 years with a disability caused by a permanent impairment [9]. People aged 65 and above have access to AT through aged care services [10]. There are also separate schemes for veterans and a range of state‐based compensation schemes for those whose disability resulted from a motor vehicle or workplace accident.

Funding schemes generally provide access to AT that is related to the person's disability, offers value for money, is not already covered by alternate services, for example, workplace schemes, and adheres to certain parameters such as the AT being considered ‘reasonable and necessary’ or important for ‘safety and independence’ [11]. Consumer‐directed models are becoming increasingly popular, where funding is allocated to a person and they choose the organisations or individual providers who will provide their services, from both the for‐profit and non‐profit sectors. Whilst it is acknowledged that this move has resulted in benefits to consumers such as access to a broader range of AT, it has also posed challenges. For instance, a major barrier to accessing AT is the extreme difficulty in navigating the funding schemes' guidelines and procedures along with making informed choices about services and products [8, 12, 13, 14]. This new approach to accessing AT has meant that people with disability are expected to be savvy consumers of AT products and services, as well as experts in funding schemes' operating guidelines [14, 15]. There are ongoing reports of poor outcomes resulting from this system, including unmet AT needs [14, 16].

Given the complexities associated with navigating funding schemes and accessing funding for AT, influenced by public policy, political realities and a focus on economic outcomes, a wide‐ranging exploration of the AT ecosystem in Queensland, Australia, was warranted. A needs assessment [17] was conducted including an audit of websites followed by interviews with representatives of the agencies and schemes that manage funding for AT (comparative and normative need) [11]; and survey and interview engagement with AT recipients (felt need) [13], and AT advisors and suppliers (expressed need) [12]. Consistent with the WHO recommendations to actively engage AT users in research (WHO 2022), the needs assessment was positioned within a participatory action research (PAR) design. The PAR approach prioritises the knowledge and experience of key stakeholders in tandem with researchers to tackle issues resulting from social systems, co‐create knowledge and devise alternatives for change [18, 19]. PAR is reflective in nature and involves genuine partnership, with balanced power between researchers and participants, who work together to understand a particular problem or issue of concern and develop actions for change [20].

Therefore, the purpose of this paper is to report the process and outcomes of patient and public involvement (PPI) as the stakeholder group for the ‘*AT Solutions for Queensland’* project across four action cycles of the PAR process that (1) designed and (2) monitored the needs assessment, and (3) generated and prioritised, and (4) finalised solutions for future work. PPI in the simplest form refers to the conduct of research ‘with’ or ‘by’ key stakeholders and is the term adopted in this study to describe the collaborative relationship and ways of working between the research team and the stakeholder group.

## 2 Methods and Results

The project emerged from discussions initiated by a representative from the National Injury Insurance Scheme Queensland (NIISQ) about a need. In response, a proposal and request for funding to support a wide‐ranging exploration and needs assessment of the AT sector in Queensland was proposed by The Hopkins Centre, and this was supported. The methods and findings of the three studies conducted during the needs assessment have been published [11, 12, 13] and two‐page summaries are freely available at https://www.hopkinscentre.edu.au/project/assistive-technology-provision-for-the-promotion-144. The focus of this paper is the stakeholder group contributions and outcomes during the development and conduct of the needs assessment and the solution generation, prioritisation and finalisation processes. Ethical approval for the project was obtained from Griffith University Ethical Review Committee (GU Ref. No. 2021/925).

### 2.1 Research Design

**Figure 1:**
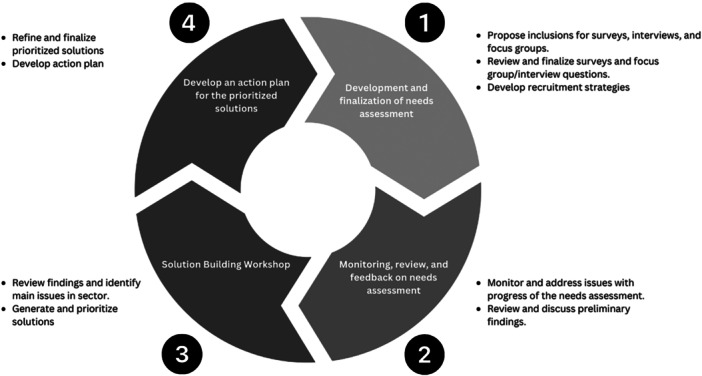
Stakeholder group involvement across the four participatory action research cycles.

A PAR approach with a series of four action cycles including the development and conduct of the needs assessment, a solution‐building workshop and solution finalisation process was completed across an 18‐month period. Figure 1 outlines the four action cycles and the involvement of the stakeholder group at each cycle. Due to the cyclical nature of the project, the methods and results of each cycle are presented concurrently in the order that they occurred.

### 2.2 Project Setting

The context of this project was the second largest state in Australia, Queensland, which has a geographical area of 1,727,000 km^2^ and a population of approximately 5,500,000 people in early 2024 [21]. One in five people lives with a disability in Queensland [22], and more than half of the population lives in regional, rural or remote areas [21].

### 2.3 Stakeholder Group

The research team have varying levels of expertise in the field of AT, and therefore it was essential that an expert stakeholder group was formed to collaborate on the codevelopment of the needs assessment plan and implementation, interrogation of the findings and to codesign solutions in response to the findings. Two AT users, who were members of the research advisory groups at The Hopkins Centre, were invited to join the stakeholder group for this study. These individuals had established working relationships with members of the research team and were invited to this study based on their lived experience and expertise in the use of a range of AT. Purposeful sampling was utilised to recruit the remaining members who would bring their unique perspective to the project as a supplier, funder or advisor of AT, and experts or organisations recognised for delivering AT‐specific services. The research team contacted stakeholders from their professional networks and invited experts from identified key organisations, for example, the national professional association of occupational therapists in Australia (Occupational Therapy Australia) and the NDIS, the largest funder of AT in Australia. Final membership of the initial stakeholder group (*n* = 14) included AT users (*n* = 2; one living with a cervical spinal cord injury and the other living with a severe traumatic brain injury), therapists experienced in providing AT services (*n* = 4), representatives from injury or disability support schemes that managed AT provision (*n* = 3), leading AT experts and researchers external to the research team (*n* = 3), a representative from a state‐wide rehabilitation community of practice (*n* = 1) and a representative from the Allied Health Professions Office of Queensland (*n* = 1). Consistent with best practice, AT users were remunerated for their time in all meetings and workshops and transport costs were covered for all attending in‐person events. The remaining stakeholder members were attending in their professional, paid capacity and did not receive any further remuneration.

### 2.4 Action Cycle 1: Study Design and Methods

The group met twice via online meetings across an initial 4‐month period. The first meeting included introductions from each group member, and ways of working were established through discussion, with a focus on the key principles of relationships first and research second and power sharing. The purpose of the project and proposed research approaches were introduced and discussed in the first meeting. Discussions identified that a mixed methods approach to survey AT users and advisors, followed by interviews or focus groups, was preferred. Recommendations for the data collection questions were recorded, and these recommendations, minutes and recordings were reviewed to inform the drafting of the questionnaires and interview/focus group guides for the project.

**Figure 2:**
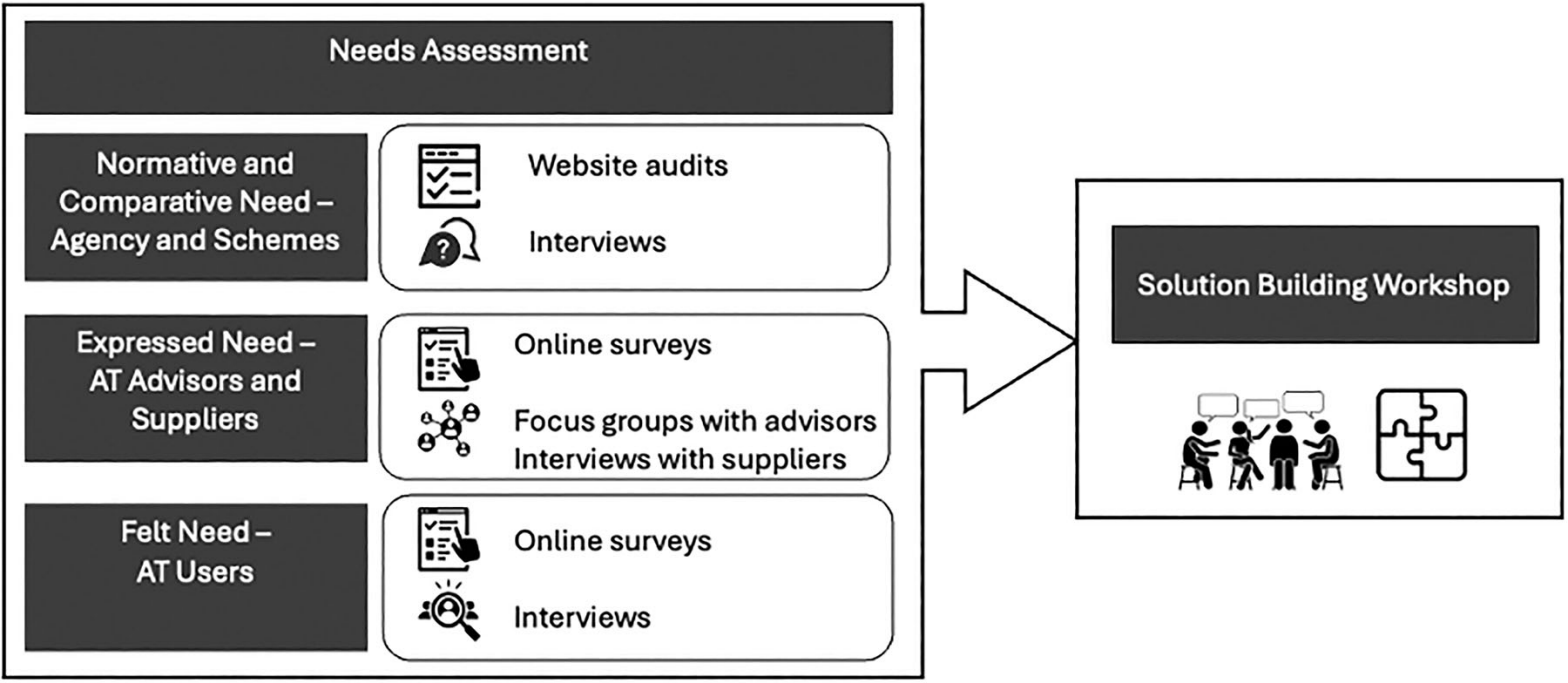
Assistive Technology Solutions for Queensland project overview.

The drafts were shared with the group before the second meeting, and the stakeholder group were advised that the focus of the second meeting was to refine and finalise the methods including recruitment and data collection questions and strategies. Feedback was collected via discussions during the meeting and email. The stakeholder group contributions were incorporated into the finalisation of the procedures of the study and the content and wording of the questionnaires and interview guides. Figure 2 outlines the finalised research design for the needs assessment and the links to the third action cycle, the solution‐building workshop. The stakeholder group level of participation in this cycle was consistent with ‘Involve’ on the International Association for Public Participation (IAP2) spectrum [23].

### 2.5 Action Cycle 2: Needs Assessment

The stakeholder group from Action Cycle 1 continued to meet bi‐monthly during the completion of the needs assessment (an additional 4‐month period) to monitor and troubleshoot the progress of the project and discuss the findings. These meetings were conducted online with an agenda and relevant documents circulated before each meeting. Early sharing and discussions of the findings of the normative and comparative need study and preliminary findings of the felt and expressed need surveys were conducted during the meetings. Examples of key outcomes from the engagement of the stakeholder group included strategies to increase survey recruitment and the addition of suppliers as a participant group in the qualitative component of the expressed need study in response to discussions of preliminary findings. The stakeholder group level of participation in this cycle was consistent with ‘Collaborate’ on the (IAP2) spectrum [23].

### 2.6 Action Cycle 3: Solution‐Building Workshop

The purpose of the solution‐building workshop was to discuss the findings from the needs assessment, the implications for the sector and to generate and prioritise solutions. The stakeholder group level of participation in this cycle was consistent with ‘Empower’ on the (IAP2) spectrum [23]. The workshop was held face‐to‐face immediately following Action Cycle 2. Members of the stakeholder group who attended the solution‐building workshop included the AT users (*n* = 2), therapists experienced in providing AT services (*n* = 4), representatives from injury or disability support schemes that managed AT provision (*n* = 3) and leading AT experts and researchers (*n* = 3). In response to feedback from the stakeholder group, one additional AT expert and two additional AT users (one with brain injury and one with spinal cord injury) were invited and attended. The representatives from a state‐wide rehabilitation community of practice (*n* = 1) and the Allied Health Professions Office of Queensland (*n* = 1) were unable to attend on the day. Therefore, a total of fifteen stakeholders attended the workshop together with six members of the research team.

#### 2.6.1 Procedure

Attendees received a booklet that summarised the main findings from the needs assessment studies before the workshop, and copies were available on the day to support participation. The stakeholders were divided into four groups, including one online group who participated in group discussions via Microsoft Teams. The four groups were purposively created to include representation from AT users, clinicians, agency/schemes and a member of the research team. The outline of the day is available as Supporting File S1.

The morning session included the presentation of the findings of the three studies. Time was allocated for questions, clarification and discussion between each presentation. The final session in the morning was a facilitated discussion of the impressions and implications of the findings from the three studies.

The afternoon session involved three rounds of solution generation and prioritisation. A ‘snowball’ or ‘pyramid’ [24] group discussion technique was applied to generate and prioritise potential solutions for the challenges discussed in the morning session. Attendees first worked within the four groups to discuss and document the solutions that they considered important for the AT sector and to rank these based on level of priority.

The four groups were then reduced to two groups (i.e., Groups 1 and 2 combined, Groups 3 and 4 combined) to share their proposed solutions and develop a combined priority list of solutions. The groups were encouraged to use the pens, highlighters, notebooks, butcher's paper and sticky notes to support their discussions and summaries of their decisions. These outputs were collected by the research team to support collation of the generated solutions and priorities before the final round.

A final, whole‐of‐group activity supported further discussion and prioritisation of the solutions. The outcome of the day was a ranked list of prioritised solutions that are outlined below. The research teams' field notes and the data collected from the notes and documentation by the attendees at the solution‐building workshop were reviewed by two members of the research team (C.H. and T.A.), and a summary of the key discussion points has been developed to build descriptions of the solutions.

#### 2.6.2 Solution‐Building Workshop Outcomes

##### 2.6.2.1 Solution 1

The main solution identified by attendees was centred on *roles for and involvement of people with lived experience in the AT ecosystem*. It was discussed that agencies and schemes, AT advisors and suppliers should work more closely with individuals with lived experience to create a system that best meets the needs of AT users. It was also highlighted that AT users were best positioned to advise on what information is helpful and how best to make this information accessible for people with disability. For example, AT users could be incorporated into the design processes of supplier and agency websites. A range of roles were discussed including the employment of people with lived experience as AT advisors or as ‘experts’, where they can provide first‐hand experience on AT use to AT users, advisors, funding agencies and suppliers. This role was discussed as potentially being particularly useful during the AT trial, for example, wherein expert AT users could provide first‐hand experience to other AT users when considering their AT options.

##### 2.6.2.2 Solution 2

*Enhanced transparency of information provided by agencies and schemes* regarding the AT processes, so that AT users and AT advisors can have a clear understanding of the expectations and the process, how decisions are made about what is approved and not approved (i.e., what would be considered reasonable and necessary) and what AT may be available to them. The information should be easily accessible and understandable to AT users and advisors.

##### 2.6.2.3 Solution 3

*Enhanced transparency of information available from suppliers of AT* to make pricing information freely and easily accessible for AT users and advisors.

##### 2.6.2.4 Solution 4

*Good practice guidelines and communities of practice* were suggested to help improve the full AT provision process, from goal setting through to follow‐up/evaluation. Good practice guides that outline the responsible parties at each step may mitigate incorrect provision, lowering the level of waste in the system. Communities of practice were recommended to support the upskilling of advisors and to enable the sharing of knowledge and resources. It was proposed that this community would increase networks of multidisciplinary experts (i.e., clinicians, people with lived experience, engineers, IT experts, data analysts, suppliers and manufacturers) and create opportunities for mentoring, where advisors could liaise with experts throughout the provision process. Peak bodies, for example, Occupational Therapy Australia, Speech Pathology Australia and/or the Australian Rehabilitation & Assistive Technology Association, were suggested as the key bodies to coordinate such communities of practice and develop good practice guidelines.

##### 2.6.2.5 Solution 5

*An individualised approach to follow‐up and evaluation* to improve the experience across the whole of the AT process. Most importantly, this meant taking an individualised approach to the training and set‐up of AT when it was received, and ongoing evaluation and review of AT over time. For improved processes, the use of telepresence was suggested to close the accessibility gap between those living in metropolitan versus rural/remote communities.

##### 2.6.2.6 Solution 6

The creation of local ‘*AT hubs*’ where people can view and trial AT in person. This solution was required to mitigate the loss of previously existing services that supported AT users to view, trial and gain expert advice on specific AT. The loss of these services has led to a situation whereby access to trial AT is now either limited or unavailable, and exponentially worse in regional and remote areas.

##### 2.6.2.7 Solution 7

A *trial to buy pathway* was suggested whereby recipients trial a piece of personalised AT through hire, and if the fit is acceptable, the option to purchase is made available.

##### 2.6.2.8 Solution 8

*Building expertise in the existing workforce* was suggested and agreed by the group to be most readily achieved through the introduction of credentialling of AT advisors (possibly managed by peak bodies).

##### 2.6.2.9 Solution 9

Lastly, *improving undergraduate education for advisors* surrounding AT was suggested as an important strategy to increase the capability of the workforce. This may include teaching what questions to ask and where to find reliable information, support and expert networks. It was agreed that the addition of formal mentoring post‐graduation or credentialling may help new graduates build their own expert knowledge regarding available AT and best practice provision.

### 2.7 Action Cycle 4: Finalisation and Action Planning

The final action cycle focused on how to action the prioritised solutions in the next phase of research and included a series of follow‐up codesign meetings across a 3‐month period with the two AT users (M.O. and H.B.) who were the original members of the stakeholder group and two members of the research team (T.A. and L.G.). These IAP2 level of participation [23] was ‘empower’ with two or three guiding questions to focus discussions, but final decision making by the AT users. AT experts (*n* = 2) were consulted throughout this phase when additional information and expertise were required to develop the solutions and next steps further. A flexible approach was taken to discussions with the AT users, based on their availability. Initially, M.O. and H.B. met separately with the lead researcher and another team member T.A. to further discuss and develop actions from the solutions. Two follow‐up meetings occurred with M.O. and H.B., and information from the AT experts was brought into these meetings for further discussion to develop and refine clear actions for the prioritised solution/s. For each of the codesign meetings, the field notes and audio recordings were reviewed by a member of the research team (T.A.), with discussion topics and action points summarised and shared with the AT users for discussion at subsequent meetings.

#### 2.7.1 Codesign Meeting Findings

During the first round of meetings, the prioritised solutions from the workshop were further discussed, and both AT users highlighted the importance of drawing from expertise in the sector and learning from best practice. It was established early that a priority solution would involve the creation of an AT hub or AT centre, which could incorporate many of the ideas from the solution‐building workshop. This included offering AT for trial, roles for AT users, and training and support for AT advisors. It could also be somewhere that develops best practice guidelines for suppliers, funders, advisors and others in the sector, and where AT users and advisors could come to learn and seek information and expertise. However, there were a number of important considerations raised by M.O. and H.B. including that it would need to offer a range of technology for display/trial so that people can learn about the options available to them; access to the hub and resources for regional and/or remote locations would need to be considered, and priority should be given to incorporating roles for people with lived experience.

In relation to building expertise in the workforce, the idea of credentialing for AT advisors was raised at these first meetings and had broad support during solution‐building workshops as a method to build capacity and expertise in the sector. Following this initial round of meetings, further information was sought from AT experts as requested by M.O. and H.B., including information on AT hubs in Australia and internationally, training currently available to AT users to work in the sector and current credentialing procedures for AT advisors in Australia. The research team reached out to two experts in the sector to gather information to inform future discussions in this phase. During the second meeting, information from these AT experts and the research team's investigations were shared including information on a range of AT hubs internationally, previous AT centres in Australia (named the Independent Living Centres), credentialing work progressed by ARATA [25], and a Certificate IV in AT peer mentoring previously available in Australia, and a programme named ATchat which partners AT users with peer mentors during the AT selection process [26]. The discussions in this second meeting focused on developing the AT hub. Discussions centred on the possibility of partnering with ATchat to offer a similar service at the AT hub in Queensland, provision of training for AT mentors at the hub either using the certificate IV developed or using this as a base to develop further training, the need for experts in specific AT devices at the hub, to be able to trial AT at the hub and linking with suppliers so a pathway to trial at home could be organised from the hub. Another idea raised was that initially, the focus of the hub should be on mid‐ to high‐level AT as complex AT is where people need the greatest support; however, information on low‐cost/low‐risk AT would still be useful. The location of the hub was also raised with a central location preferred in a major Queensland city, likely Brisbane, with easy transport and access required. The need for the hub services to be available to all users was also raised as an important consideration, this included those in hospital, rehabilitation and living in the community, and who were accessing AT through a range of schemes.

After this second meeting with M.O. and H.B., it was agreed that T.A. would develop a proposed research plan for the next stage to develop and evaluate an AT hub in Brisbane, Australia, along with two further initiatives to address the prioritised strategies. The first initiative, the AT hub, proposed the inclusion of training of and access to AT users as AT mentors; training, resources and access to specialists to mentor and support AT advisors; integration with suppliers, and a focus on complex or high‐risk AT initially. The second initiative focused on the development and trial of AT mentor training, and the third suggested the development and trial of a training and credentialing system for AT advisors. Both M.O. and H.B. strongly supported the proposed plan, with suggestions on how to improve the proposal. This included the need to involve a wide group of people with lived experience of AT use in the research moving forward, which could be done by partnering with a group of lived experience experts. They also suggested to broaden the range of stakeholders to partner with to include professional associations and previous AT centres across Australia, which still operate, while at a reduced capacity. There was also a suggestion that it would be useful if the hub could incorporate information about how to access or methods to enable access to recycled or second‐hand AT to reduce waste in the sector.

## 3 Discussion

This paper reports a programme of participatory research that partnered with a stakeholder group to conduct a needs assessment of the AT sector in one geographical region and collaboratively generate context‐specific solutions and actions for the future. It is important to recognise that many of the identified issues, solutions and actions to emerge from this programme of research are not new and have been previously promoted by WHO [4, 27]. Indeed, a recent scoping review exploring AT barriers in the World Health Organization (WHO) European region identified three key barriers to accessing AT: affordability, acceptability and access, which relate to systemic failures. These systemic failures included fragmented systems, as well as a lack of knowledge and awareness among health and social care staff, a lack of training and evidence to support AT, inadequate assessment procedures, a lack of collaborative relationships between users and health and social care staff, and a lack of policies and dedicated government bodies for AT [28]. The contribution of the research reported in this manuscript is the prominent roles of the members of the stakeholder group through all participatory action cycles and the privileging of their ideas and perspectives in the solution to action cycles. In this discussion, we consider the three recommended actions from the project within the local and global AT landscape and how the stakeholder group (PPI) involvement impacted the outcomes of the project.

The first recommended action was to develop an AT hub that would be a centre of expertise where AT users and advisors could come to seek and learn information. A range of strategies were considered important for the hub's success including availability of AT to view and trial, linking with suppliers for longer term trial, access to AT mentors, and training and support for AT advisors. It is important to acknowledge that AT hubs or AT centres, where a range of AT is available to trial as well as information and education for users and AT advisors, have existed for decades. Indeed, they previously existed within the Australian context in the form of Independent Living Centres but have closed or made significant changes to their business models in response to changes in funding models. A federal government joint standing committee inquiry into the provision of AT under the NDIS [29] warned of the potential loss of these state‐funded centres, foretelling that the independent advice and the ability to trial AT in a neutral environment would likely be lost. This warning was not heeded, and the solutions and actions generated by the stakeholder group highlight that previous policy changes towards fee‐for‐service models have had a detrimental impact on the Australian AT sector.

From a global perspective, the Bologna Declaration of the Association for the Advancement of Assistive Technology in Europe [30] states that AT systems should be person‐centred and independent from commercial interests. The development of innovative service models such as mobile clinics, home or community‐based services or tele‐rehabilitation that are specific to the local context has been championed [4]. Examples include the Assistive Devices Centre in Taiwan which offers AT demonstration, loans, maintenance, consultation and evaluation [31], and in the United Kingdom, the new online AT centre, Living Made Easy, offers impartial advice and information on AT, as well as training and resources for AT advisors [32]. The importance of the impartial information and advice that was previously provided by the independent living centres in Australia has been acknowledged by the government, and the centres have been promoted as best practice for the trial of AT [33, 34].

The second recommended action aligns with the research priority of the WHO to invest in the involvement of AT users and groups to bridge the gap in AT personnel worldwide [27]. Notably, the stakeholder group identified and privileged the role of AT users beyond that of simply consumers of services and recommended that they should be involved at all levels of the AT sector. AT mentor training was prioritised as an actionable solution, and there are examples of this approach from around the world such as a pilot peer‐led wheelchair training programme in Canada which significantly improved manual wheelchair users' self‐efficacy in community living and their skills in wheelchair use [35], and a team of AT mentors in the United Kingdom who use augmentative and alternative communication to support people with their communication goals (https://www.attherapy.co.uk/at-mentors). The AT chat model is an AT mentor website in Australia (https://www.atchat.com.au/) that has found positive results with AT mentors supporting access to information unbiased by financial interests, promoting choice and control in AT decision making and mentees reporting improved self‐efficacy and feeling empowered to conduct their own information gathering and choice‐making in the future [36]. However, current barriers to AT mentor workforce growth in Australia include the lack of a professional registration body to support safe and high‐quality services, a lack of viable funding streams and the urgent need for a professional qualification, with the Certificate IV in AT mentoring no longer available (personal communication, 2023 Natasha Layton, Independent Chair of the National Assistive Technology Alliance [NATA]). Without addressing such barriers, the viability and sustainability of such resources as ATchat may be challenged. ARATA (www.arata.org.au) have recently launched an online micro‐credential titled ‘An introduction to the good practice steps of assistive technology provision’ for AT users, advisors, suppliers, researchers and educators, which in part may address this need.

The third recommended action was the training and credentialing of AT advisors. There is an urgent need to improve workforce capacity globally [4, 27] and the need for the establishment of systems to provide evidence‐based assessment of AT advisors' competence has long been felt in Australia. The World Health Organization offers an online Training in Assistive Products programme (https://www.who.int/teams/health-product-policy-and-standards/assistive-and-medical-technology/assistive-technology/training-in-products), which supports a range of personnel to develop knowledge and skills in the safe and effective provision of basic AT worldwide. In the lead up to the introduction of the NDIS, ARATA developed an options paper recommending additional credentialing beyond professional registration for high‐risk AT with several pathways suggested including professional qualifications, certificate III plus experience or a minimum of 3 years equivalent full‐time experience [8]. A recent scoping review has established guidelines for AT service provision including steps and a quality framework [37]. However, training is important for ensuring that AT advisors are skilled in the facilitation of the essential initial steps of AT provision, namely the provision of information on what AT is available, exploration of the possibilities AT can offer, and the trialling of AT to support informed decision making [38, 39, 40]. If provided with these opportunities, it is more likely that the most appropriate AT is selected for the individual, reducing abandonment and non‐use. It is therefore critical that there is a research agenda that not only evidence the value of AT services in the form of AT hubs [27] but establishes a pathway that targets ongoing sustainability of an AT training and credentialing system, beyond research funding cycles.

The PAR approach and the collaboration with a stakeholder group across the lifespan of the project greatly enriched the processes and findings beyond that which would have been found with the needs assessment [11, 12, 13]. The increasing and prolonged engagement in the solution and action‐building processes ensured that potential bias from one voice or perspective was minimised. The involvement of the stakeholder group and in particular the AT users ensured that *people* remained central to discussions as promoted by the WHO's people‐centred AT ecosystem framework [6]. The recommended actions address the *personnel* and *provision* aspects of the framework and provide a clear direction for the AT Sector in Queensland. We have reflected that the increasing level of participation, from involve through to empower, was a strength of the project, but that it would have been preferable to ensure increased collaboration from the project's inception. Notably, it is essential that the next steps towards implementation are conducted with continuing partnerships and with increased recognition of the possible systemic constraints and enablers. In particular, the privileging of the voices of AT users in the final action cycle, although considered a strength of this project, does mean that additional processes are necessary to codesign detailed implementation plans.

## 4 Limitations

There are some limitations to the design to note, including the mix of the stakeholders involved in the stakeholder group, workshop and codesign meetings. AT suppliers were not represented in the stakeholder group, and their inclusion may have led to more robust and critical considerations with regard to strategies for AT trial and supply. There was also a small number of AT users involved in the stakeholder group, workshop and codesign meetings, which was raised by those AT users as a concern and that a greater diversity of AT users would be needed in the implementation of the strategies to ensure they are appropriate for a breadth of needs. The needs of regional and remote communities were raised at several points throughout the project; however, the lack of any regional and remote representation in the project stakeholder group limited deeper discussion and focus on the needs of those in regional and remote areas. These limitations highlight the importance of continuing work by the team expanding the representation of stakeholder groups as coresearchers and on advisory or stakeholder groups when designing, implementing, and evaluating the impact of the codesigned initiatives. Finally, the project was specific to the AT sector in one geographical area, and therefore the findings are specific to that region and will not represent the diverse contexts worldwide.

## 5 Conclusion

This project sought to partner with a stakeholder group to identify needs and codesign solutions and actions for the AT sector in Queensland. Three actions were proposed following four cycles of PAR: the development of an AT hub, AT mentor training, and a training and credentialing system for AT advisors. The hub was prioritised as it allows for a range of needs to be addressed, incorporating roles for AT users, and AT advisor skill and expertise development. Learnings from the partnership with the stakeholder group highlight the need to involve a diverse AT user group in the initiative's implementation and partnering with experts and resources already created within the sector. The three initiatives address the provision and personnel aspects of the WHO's people‐centred AT ecosystem framework [6]; however, for the initiative to result in ongoing success, the policy environment must support these initiatives. As such, in re‐establishing or creating AT hubs or centres, evidencing their value will be essential; cost‐effectiveness studies would therefore be warranted to support their ongoing funding and expansion. In line with this, work to develop and trial a training and credentialing system for AT advisors must consider its ongoing sustainability to be successful.

## Author Contributions

Tammy Aplin led the participatory processes outlined in Action Cycle 4, drafted and finalised this manuscript and approved it for submission. Louise Gustafsson led the needs assessment, solution‐building workshop and participatory processes outlined in Cycles 1–3. She participated in the participatory processes outlined in Cycle 4 and has contributed to the development and finalisation of this manuscript and approved it for submission. Michelle Owens and Hayden Boyd were members of the stakeholder group throughout all action cycles. They have contributed to the finalisation of this manuscript and approved it for submission. Tenelle Hodson, Camila Shirota, Christy Hogan and Michelle Bissett were involved in the needs assessment, solution‐building workshop and participatory processes associated with Cycles 1–3. They have contributed to the finalisation of this paper and approved it for submission.

## Ethics Statement

Ethical approval was granted for the study by Griffith University (GU Ref. No. 2021/925).

## Conflicts of Interest

The authors declare no conflicts of interest.

## Supporting information

Supporting Information.

## Data Availability

The authors have nothing to report.
