# Supplementary figures and images for: Codesigning Solutions for Assistive Technology Service Provision in Queensland, Australia
Source: Health Expect. 2025 Jun 6;28(3):e70322. doi: 10.1111/hex.70322 (PMC12141917; doi:10.1111/hex.70322)

Supplementary File One

Solution Building Workshop Outline


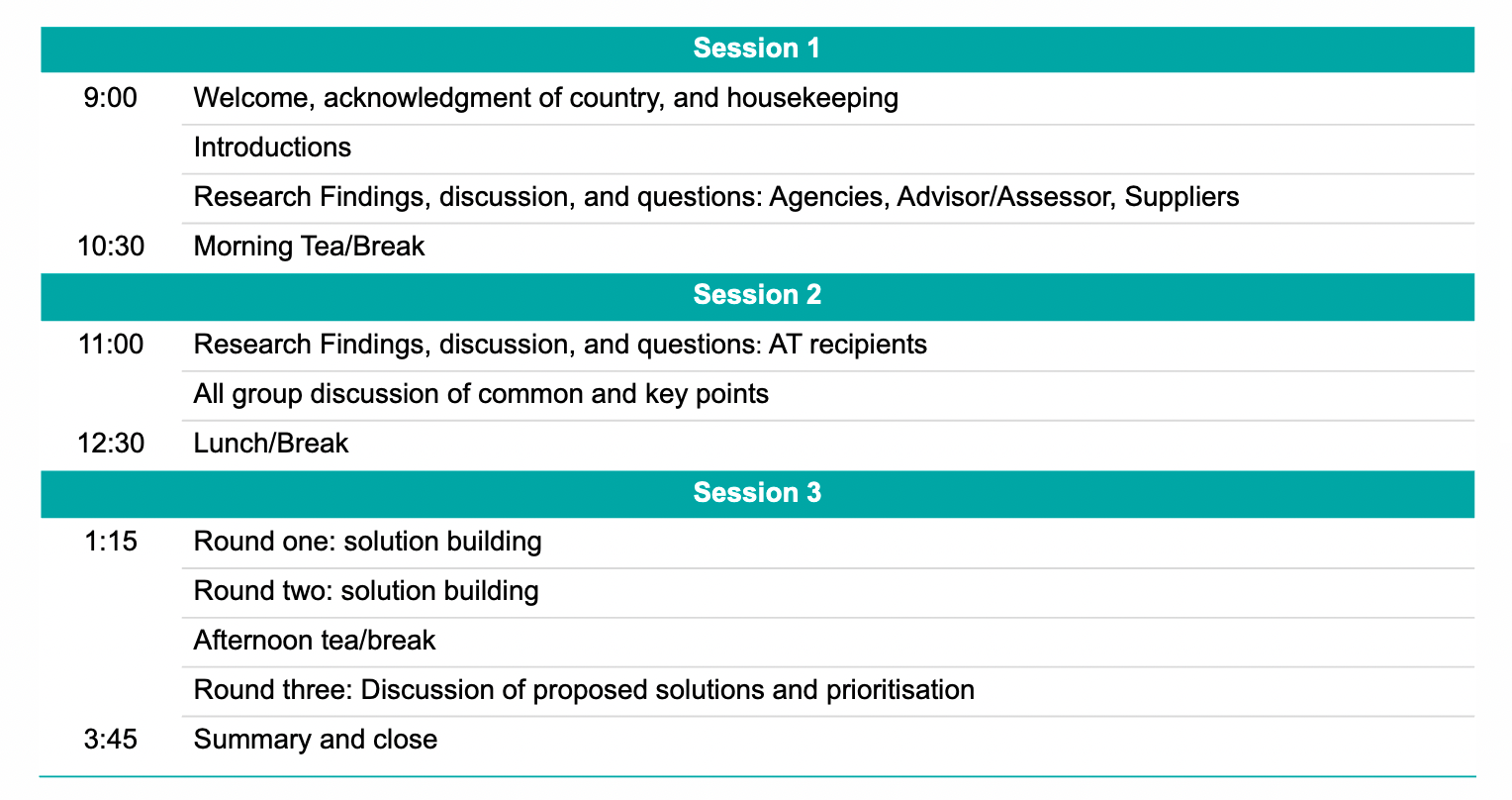

Supplement: Supplementary file 1 — Supporting Information. [file HEX-28-e70322-s001.docx]
